# Accelerated partial breast irradiation: advances and controversies

**DOI:** 10.1186/s40880-016-0095-1

**Published:** 2016-03-24

**Authors:** Mani Akhtari, Bin S. Teh

**Affiliations:** Department of Radiation Oncology, Houston Methodist Hospital, Cancer Center and Research Institute, Weil Cornell Medical College, 6565 Fannin, Ste#DB1-077, Houston, TX 77030 USA; Department of Radiation Oncology, The University of Texas Medical Branch at Galveston, Houston, TX 77555 USA

**Keywords:** Accelerated partial breast irradiation, Brachytherapy, Breast cancer

## Abstract

The management of localized breast cancer has changed dramatically over the past three to four decades. Breast-conserving therapy, which involved lumpectomy followed by adjuvant irradiation, is now widely considered the standard of care in women with early-stage breast cancer. Accelerated partial breast irradiation (APBI), which involves focal irradiation of the lumpectomy cavity over a short period of time, has developed over the past two decades as an alternative to whole breast irradiation (WBI). Multiple APBI modalities have been developed including brachytherapy, external beam irradiation, and intraoperative irradiation. These new techniques have provided early-stage breast cancer patients with shorter treatment duration and more focused irradiation, delivering very high biological doses to the region at a high risk of failures over a much shorter treatment course as compared with conventional radiotherapy. However, the advantages of APBI over conventional radiotherapy are controversial, including a higher risk of complications reported in retrospective literature and shorter follow-up duration in the intraoperative APBI trials. Nevertheless, APBI presents a valuable alternative to WBI for a selected population of women with early-stage breast cancer.

## Background

The management of localized breast cancer has changed dramatically over the past three to four decades. Mastectomy, the initial management of this disease, has shifted increasingly towards breast-conserving therapies (BCT) after landmark trials such as the National Surgical Adjuvant Breast and Bowel Project B-06 [1], Milan [2], and the European Organization for Research and Treatment of Cancer 10801 [3] showed that mastectomy was equivalent to breast-conserving surgery (BCS) followed by postoperative irradiation. In these trials, the addition of whole breast irradiation (WBI) after BCS decreased the chance of ipsilateral breast tumor recurrence by 50% or more, indicating the need for radiotherapy to address any residual microscopic disease. However, WBI usually consists of 5–6 weeks of daily irradiation, which can impose certain logistical, economical, and social barriers to care. In fact, in women who undergo BCS, the percentage of patients who undergo radiotherapy can vary from 12% to 77% depending on their age, access to a radiation therapy center, and comorbidities [4]. Additionally, 68% of ipsilateral breast tumor recurrences are within 3 cm from the primary tumor, emphasizing the need to focus around the area of the initial disease and raising the necessity of irradiation on the whole breast of patients with early-stage breast cancer. This has led to development of techniques that shorten the courses of radiation to the immediate area surrounding the tumor bed. Due to the decreased number of irradiation fractions, shortened treatment duration, and decreased size of the irradiation field, this group of treatments is collectively named accelerated partial breast irradiation (APBI).

## Brachytherapy in APBI

One of the most widely used APBI approaches is via brachytherapy (B-APBI), which involves placement of radioactive sources into the breast tissue to deliver high doses of radiation to a confined area. Here we discuss the different forms of B-APBI and the existing data justifying their safety and efficacy.

### Interstitial brachytherapy

Interstitial brachytherapy is the first technique developed and used to treat only a partial amount of breast tissue. Although the first published series of interstitial brachytherapy date back to 1920s, breast interstitial brachytherapy did not come into prominence until the 1970s [5]. At that time, BCT was being developed, and since no electron beam therapy was available, a boost was given to the vicinity of the tumor bed using low-dose-rate (LDR) interstitial brachytherapy. With the advent of high-energy linear accelerators, electron beam boosts for the most part supplanted interstitial brachytherapy. However, in the meantime, brachytherapy techniques also improved dramatically along with better dose homogeneity, resulting in improved overall cosmesis [6]. As the experience with interstitial brachytherapy for boost grew, several trials were undertaken to evaluate its safety and efficacy as the sole modality of irradiation after BCS.

To date, numerous single-arm and some randomized studies have been conducted examining multi-catheter interstitial brachytherapy. The results of the most important trials are summarized in Table 1 [7–14]. Most of these studies enrolled patients with early-stage breast cancer, T1 or T2, with some allowing up to three positive axillary lymph nodes (N1). All studies required negative surgical margins and the majority of the trials excluded women with lobular or ductal carcinoma in situ (LCIS or DCIS) histology. Interstitial catheters were placed anywhere from 4 to 8 weeks after surgery using either a free-hand technique or a breast template with the placed surgical clips as a point of guidance. Some of the later studies used 3-dimensional planning. Consistent with the general trends in overall use of brachytherapy, a percentage of earlier patients were treated with LDR or pulsed-dose-rate (PDR) sources, but the majority of the more recent patients were treated using ^192^Iridium (^192^Ir) high-dose-rate (HDR) brachytherapy. In almost all of the studies, the tumor bed plus 2 cm (some 1–2 cm) was covered by the radiation. LDR doses ranged from 45 to 50 Gy and HDR from 30 to 36 Gy using twice daily (BID) fractionation. As seen in Table 2 [15–17], with careful patient selection, ipsilateral breast tumor recurrence rates were very low except for that in the Guy’s Hospital trial by Fentiman et al. [13], which reported an ipsilateral breast tumor recurrence rate of 18%. The overall cosmesis scores were good to excellent for the majority of the patients with low rates of late complications. Given the promising results of these and earlier studies, APBI slowly became an acceptable option for a limited population of patients with early-stage breast cancer. Its widespread use was however limited by the complicated insertion technique of interstitial brachytherapy catheters.

**Table 1:** Results of randomized and single-arm interstitial brachytherapy trials

Reported trial	Number of patients	Treatment volume	Source/dose	Median follow-up (months)	Ipsilateral recurrence rate (%)	Outcome/complications
Wazer et al. [7]	32	Excision cavity + 2 cm	^192^Ir, 3.4 Gy BID to 34 Gy	33	3	8 with fat necrosis, 11 with grade 3–4 skin toxicity
Arthur et al. [8]	44 (31 HDR, 13 LDR)	Lumpectomy cavity + 2 cm	^192^Ir, HDR: 3.4 Gy BID to 24 Gy; LDR: 50 cGy/h to 45 Gy	42	0	43% of LDR patients had radiation recall with adriamycin
Benitez et al. [9]	199	Lumpectomy bed + 1–2 cm	LDR ^125^I, 0.52 Gy/h to 50 Gy; HDR ^192^Ir, 3.2-3.4 Gy BID to 32–34 Gy	68.4	1.2	11% fat necrosis, 90% good-excellent cosmesis
Ott et al. [10–12]	274	Tumor bed + 2 cm	^192^Ir, PDR: at 0.6 Gy pulses to 50 Gy; HDR: 4 Gy BID to 32 Gy	63	2.9	2.6% ≥ grade 3 toxicity, 90% good to excellent cosmesis
Fentiman et al. [13]	50	Tumor bed + 2 cm	^137^Cs, 4 fractions, 4–6 h/day to 45 Gy	75.6	18	82% good to excellent cosmesis
Polgár et al. [14]	45	Tumor bed + 1–2 cm	HDR ^192^Ir, 7 fractions of 4.33 or 5.2 Gy in 4 days to 30.3–36.4 Gy	133	8.9	77.8% with good to excellent cosmesis, 2.2% with fat necrosis

*BID* twice daily, *HDR* high-dose-rate, *LDR* low-dose-rate, *PDR* pulsed-dose-rate

**Table 2:** Patient selection criteria for accelerated partial breast irradiation (APBI)

Organization	Age (years)	Tumor size (cm)	Lymph node status	LVSI	Margin	Multifocality	DCIS	Neoadjuvant therapy	Histology
ASTRO [15]									
Suitable	≥60	≤2	pN0 (i^+^/i^−^)	No	Negative (≥2 mm)	Clinically unifocal	None	None	IDC
Cautionary	50–59	2.1–3.0	–	Limited/focal	Close (<2 mm)	Clinically unifocal	≤3 cm	–	ILC
Unsuitable	<50	>3	≥pN1	Extensive	Positive	Multifocal	>3 cm	Used	–
ASBS [16]	≥45	≤3	N0	–	Negative	–	≤3 cm	–	IDC or DCIS
ABS [17]	≥50	≤3	N0	–	–	Unifocal	–	–	IDC

*LVSI* lymphovascular space invasion, *DCIS* ductal carcinoma in situ, *ASTRO* American Society of Therapeutic Radiation Oncology, *pNi* pathologically positive node determined by immunohistochemistry with a size ≤ 0.2 mm, *IDC* invasive ductal carcinoma, *ILC* invasive lobular carcinoma, – not mentioned, *ASBS* American Society of Breast Surgeons, *ABS* American Brachytherapy Society

### Single- and multi-lumen applicators

**Fig. 1:**
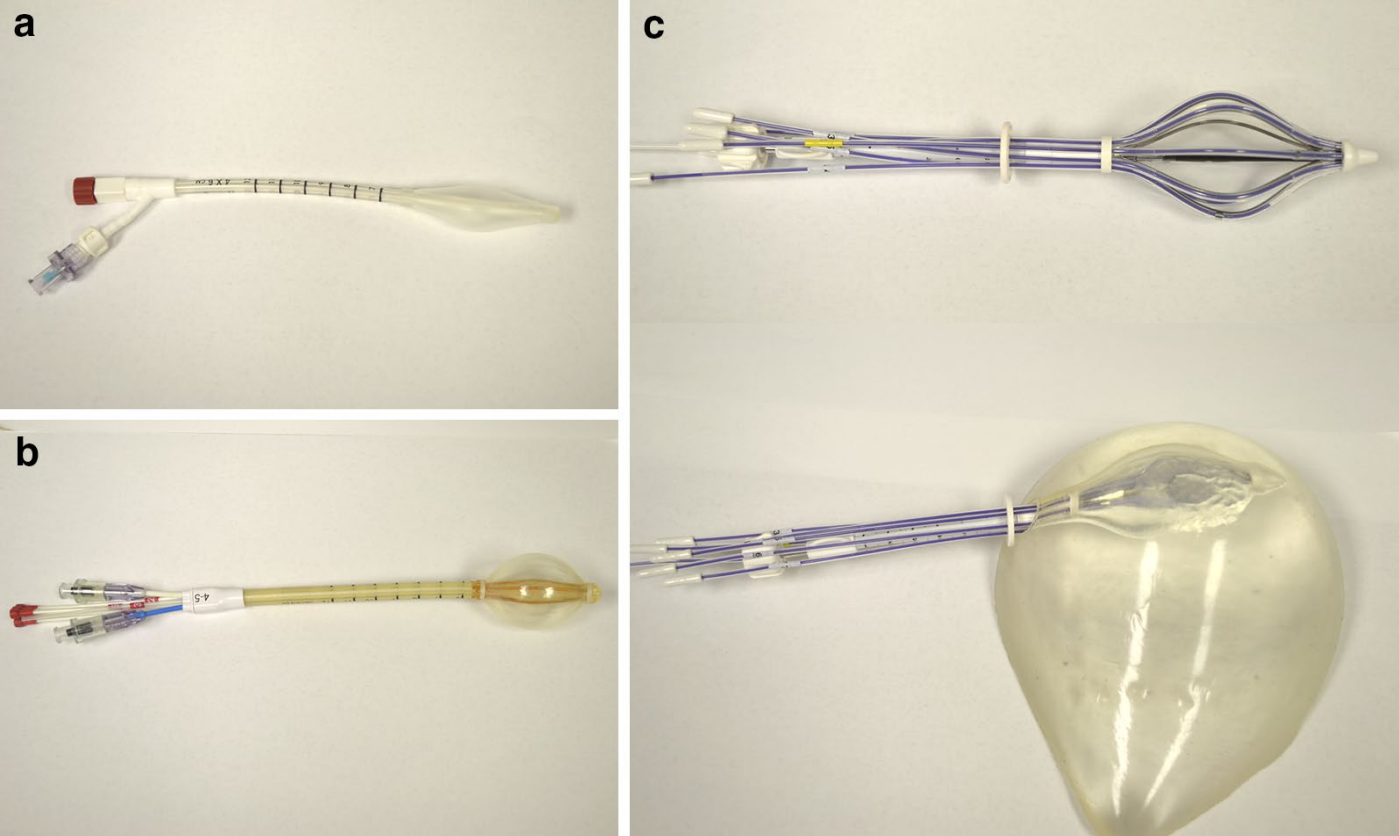
Single- and multi-lumen/multi-catheter applicators used in accelerated partial breast irradiation. **a** An inflated 4X6 MammoSite^®^ (Hologic, Bedford, MA, USA) balloon. **b** An inflated 4–5 Contura^®^ (SenoRx, Inc., Aliso Viejo, CA, USA) balloon. **c** An expanded SAVI^®^ (Cianna Medical, Aliso Viejo, CA, USA) 8–1 balloon (*top*) and placed in plastic model (*bottom*)

In 2002, the Food and Drug Administration (FDA) of the United States approved the MammoSite^®^ (Hologic, Bedford, MA, USA) balloon applicator, the first of its kind that simplified catheter insertion and usage of APBI compared with the placement of catheters in interstitial brachytherapy (Fig. 1a). Between 2002 and 2004, 97 institutions participated in a registry trial, which was designed to collect data on the use of MammoSite^®^. Collectively named the American Society of Breast Surgeons (ASBS) Registry Trial, a total of 1449 patients with early-stage breast cancer were treated using 34 Gy in 10 BID fractions. At a median follow-up of 60 months, only 2.6% of the patients developed an ipsilateral breast tumor recurrence, and 90.6% had good or excellent cosmesis score. The rates of complications were low with a reported 2.3% of fat necrosis and 13% of symptomatic seromas [18]. In a separate analysis, the rate of axillary failure in the same registry of patients was 0.79% with a 5-year overall survival (OS) rate of 77.8% in patients with an axillary failure [19]. Additionally, 194 of the patients in the MammoSite^®^ registry trial were treated with a diagnosis of DCIS. Long-term follow-up with a median of 54.4 months showed only a 3.1% rate of ipsilateral breast tumor recurrence [20].

As MammoSite^®^ became increasingly popular, some of its shortcomings also became apparent. Given its single-lumen catheter, dosimetric shaping and conformation to the surrounding tissue can be challenging. This led to the development of other multi-lumen balloon-based catheters, including MammoSite ML^®^ (Hologic, Bedford, MA, USA) and Contura^®^ (SenoRx, Inc., Aliso Viejo, CA, USA). As opposed to the original MammoSite^®^ balloon, which included a single lumen surrounded by an inflatable balloon, the Contura^®^ balloon includes a central catheter and four surrounding fixed struts (Fig. 1b). Several dosimetric studies quickly showed lower dose delivered to organs at risk (OARs) using multi-lumen applicators than using single-lumen applicators. Cuttino et al. [21] compared the dose to the skin and chest wall for 43 patients treated with interstitial multicatheter technique, 45 treated with Contura^®^, and 83 with MammoSite^®^ and observed higher maximum skin and rib doses by MammoSite^®^ compared with those by the multi-lumen techniques. In another study, Brown et al. [22] compared 33 patients treated with Contura^®^ with 33 patients treated with MammoSite^®^. Despite closer skin spacing, the Contura^®^ technique showed lower median skin dose, lower rib dose, and equal or better planning target volume (PTV) coverage as compared with the MammoSite^®^ technique. Clinical outcome data using MammoSite^®^ have also been promising, with a single-institution series reporting an ipsilateral breast tumor recurrence rate of 2%, no grade 3–4 toxicities, and good/excellent cosmetic outcome in 97% of 46 patients at a median follow-up of 36 months [23].

To allow even more dosimetric flexibility, Strut Adjusted Volume Implant (SAVI^®^, Cianna Medical, Aliso Viejo, CA, USA) was developed which included a central catheter surrounded by 6, 8, or 10 peripheral catheters (Fig. 1c). Initial results using SAVI^®^ have been excellent dosimetrically, with evaluation of 102 patients treated revealing target volume receiving 90% of prescribed dose (V90) of 95.9% and maximum median skin dose of 75% of prescribed dose [24]. At a median follow-up of 21 months, the ipsilateral breast tumor recurrence rate was 1%, and the rate of symptomatic seroma or fat necrosis was 1.9%, showing low rates of toxicity and recurrence in carefully selected patients consistent with other B-APBI data.

### Technique and catheter placement

Since the use of multi-catheter interstitial brachytherapy has declined dramatically in the recent past and is only reserved to few specialized centers, this section will instead focus on placement of single- and multi-lumen applicators (MammoSite^®^, Contura^®^, and SAVI^®^). Initial techniques for insertion of the MammoSite^®^ balloon required placement either at the time of lumpectomy (open-cavity technique) or as a separate procedure (closed-cavity technique) up to 10 weeks after operation. If placed postoperatively, the device can then be inserted either through the surgical scar or through a separate incision. Ultrasound guidance is frequently used to detect the seroma, which in most cases associates with the actual tumor bed and aligns the route of insertion along the longest axis diameter of the cavity. Some institutions obtain a computed tomography (CT) scan several days before insertion of the catheter to measure the size of the cavity and estimate the needed device size. After insertion, the balloon is then inflated with sterile saline to a diameter of 4.0–5.0 cm, and a small amount of radiographic material is added for contrast enhancement. A CT scan is then obtained after placement of the balloon to evaluate the conformance of the balloon to the cavity and absence of air or fluid gaps. A ratio of air or fluid in the cavity to balloon surface of less than 10% is usually deemed acceptable, and a balloon-skin distance equal or greater than 5 mm is ideal. The lumpectomy cavity is then delineated and expanded by 1 cm to define the PTV. Each expansion and PTV should conform to the patient’s anatomy, stay 5 mm from the skin, and remain at the edge of the pectoralis muscles and ribs. The most commonly prescribed dose is 3.4 Gy BID to a total of 34 Gy. Although normal tissue constraints can vary from institution to institution, the maximum allowable skin dose is kept below 100% of the prescription. If the balloon-skin distance is 5–7 mm, up to 145% of the prescribed dose is also acceptable. Ideally, 95% of the PTV should receive 95% of the prescribed dose, and V150 and V200 (volumes of tissue receiving 150% and 200% prescribed dose) should be limited to 50 and 20 cc, respectively. It is recommended that conformance and balloon-skin distance be assessed daily before each treatment session. The placement and dosimetric constraints of the Contura^®^ and SAVI^®^ balloons follow a similar protocol.

## External beam APBI

As B-APBI was slowly growing in popularity, its use was still limited to centers with brachytherapy expertise and access to LDR or HDR afterloaders. If the rationale for B-APBI holds true that in the majority of early-stage breast cancer patients the risk of recurrence is limited to the area surrounding the lumpectomy cavity, then the same should hold true for treatment using external beam. Nevertheless, external beam APBI (EB-APBI) is not a relatively new concept, and some of the earliest trials using EB-APBI date back to as early as 1980s. Ribeiro et al. [25] randomized 708 patients who were treated at Christie Hospital between 1982 and 1987 to undergo WBI to 40 Gy in 15 fractions or tumor bed-only irradiation using electrons to 40–42.5 Gy in eight fractions. After a median follow-up of 5.4 years, the ipsilateral breast tumor recurrence rate was 15%–34% for patients who underwent limited field irradiation compared with 8%–14% for patients who underwent WBI depending on the pathology. They noted that even though limited field electron irradiation was feasible, the local recurrence rate was higher than that of patients who underwent WBI, and better patient selection and treatment techniques were needed to improve outcomes. In another similar study, Dodwell et al. [26] randomized patients to undergo WBI to 40 Gy in 15 fractions or APBI to 55 Gy in 20 fractions using electrons. The study accrued only 174 out of expected 400 patients and closed early due to non-accrual. Although this limited any definitive conclusions that can be drawn from this study, the differences in the rates of ipsilateral breast tumor recurrence (WBI 4% vs. APBI 12%), locoregional failure (WBI 24% vs. APBI 9%), distant metastases (WBI 27% vs. APBI 23%), or OS (WBI 73% vs. APBI 70%) were not significant.

More recently, the most significant EB-APBI trial to date, named the Randomized Trial of Accelerated Partial Breast Irradiation (RAPID), released its interim cosmesis and toxicity analysis, showing higher rates of adverse cosmesis and late radiation toxicity in the EB-APBI group compared with the WBI group [27]. In this study, 2135 patients with invasive ductal carcinoma (IDC) or DCIS with tumor size ≤3 cm, negative surgical margins, negative nodes determined by pathologic assessment, and older than 40 years were randomized to either WBI to 42.5 Gy in 16 fractions or 50 Gy in 25 fractions or EB-APBI to 38.5 Gy in 10 BID fractions of 3.85 Gy. In the WBI group, 21% of the patients received boost to the tumor bed, and none in the EB-APBI group. After a median follow-up of 3 years, the EB-APBI group showed worse cosmetic outcome assessed either by trial nurse (33% vs. 13% in the WBI group) or by patient self-assessment (32 vs. 21 % in the WBI group). The rates of grade 1–2 toxicity were higher in the EB-APBI group than in the WBI group. Although other non-randomized single- and multi-institution experiences from William Beaumont [28], RTOG 0319 [29], and Rocky Mountain Cancer Center [30] have shown good local control and cosmetic outcome, the results of the RAPID trial [27] have added to previous experiences from Tufts University [31] and University of Michigan [32] that had called into question the unacceptable rates of cosmesis and toxicity using EB-APBI. Therefore, at this point we do not recommend using EB-APBI outside of the settings of a clinical protocol.

## Intraoperative APBI

Intraoperative APBI (IO-APBI) has been a new and exciting development in APBI. The two most extensively studied devices capable of delivering IO-APBI are the Intrabeam^®^ device (Oberkochen, Germany) and the Novac7^®^ device (Hitesys, Latina, Italy). Intrabeam^®^ produces low-energy photons up to 50 kV using spherical applicators of varying sizes with intraoperative treatment duration of approximately 30 min. In the recently updated targeted radiation therapy trial (TARGIT-A), an international cohort of participants enrolled 3451 patients who were randomized to either conventional WBI per each center’s protocols or IO-APBI using Intrabeam^®^, with a single 20 Gy fraction immediately after lumpectomy prescribed to the surface of the applicator (about 5–7 Gy at 1 cm) [33]. If the patients had pre-defined adverse pathologic features including LCIS, lymphovascular space invasion, positive nodal status, or other parameters defined at each center, postoperative WBI was added, and the IO-APBI was counted as the boost. With a primary endpoint of local recurrence, the non-inferiority of IO-APBI was set at a 2.5% absolute difference in local recurrence. At a median follow-up of 2 years and 5 months, the IO-APBI group showed a local recurrence rate of 3.3% vs. 1.3% in the WBI group (*P* = 0.04), meeting the non-inferiority criteria. Additionally, the rates of OS or distant metastases were not significantly different, and the IO-APBI group also showed low rates of grade 3–4 skin toxicity.

The Novac7^®^ device has been evaluated in the Milan Electron IntraOperative Trial (ELIOT) study [34]. The device is a mobile accelerator capable of generating different electron energies ranging from 4 to 12 MeV. In this trial, 1305 patients were randomized either to conventional WBI or to receive a single fraction of intraoperative electrons to a dose of 21 Gy prescribed to the tumor bed. The target was surgically constructed, and the thoracic shielding was placed underneath the target. The total delivery duration was 30–40 min. Inclusion criteria included age >48 years and tumor diameter ≤2.5 cm in women who were eligible for BCS. After a median follow-up of 5.8 years, 35 patients in the IO-APBI group and 4 in the WBI group experienced ipsilateral breast tumor recurrence (*P* < 0.0001), equaling 5-year event rates of 4.4% vs. 0.4%. The OS rate was similar between the two groups and the rate of skin adverse effects was significantly lower in the IO-APBI group than in the WBI group (*P* = 0.0002).

Although the above IO-APBI trials show some promising early results, the follow-up for both trials is relatively short especially given that breast cancer can recur many years later. Other logistical issues also exist such as limited knowledge of tumor pathology at the time of the surgery and high local recurrence rates seen in the ELIOT study. Additionally, the patients in these trials were highly selected, and it is unclear if IO-APBI can be considered a therapeutic option in any other group of early-stage breast cancer patients. Nevertheless, these studies have paved the way for future investigations into the use of IO-APBI. Given that it is extremely convenient for the patients and delivered in a single fraction at the time of the surgery, IO-APBI could be a reasonable option for a small subpopulation of early-stage breast cancer patients.

## Selection criteria

To date, there have been no randomized trials comparing WBI with B-APBI. As such, specific criteria that are deemed universally acceptable for patients who are appropriate for B-APBI are not known. In the early days of interstitial APBI, since the criteria had not yet been determined, the majority of patients treated were those with early-stage breast cancer. This trend continued to the era of single- and multi-lumen applicators. Through many years of cumulative experience, certain trends have started to emerge about which group of patients can safely undergo B-APBI. Therefore, professional societies, including the American Society of Therapeutic Radiation Oncology (ASTRO) [15], the American Society of Breast Surgeons (ASBS) [16], and the American Brachytherapy Society (ABS) [17], have put forward their own specific recommendations about which patients can be safely treated with APBI (Table 2). Of note, these recommendations apply to all forms of APBI, including EB-APBI and IO-APBI, which will be discussed below. As can be seen from Table 2, ASTRO recommendations are divided into three categories labeled “suitable,” “cautionary,” and “unsuitable”. We recommend only treating patients under the suitable or those limited to one or two cautionary features off of a randomized protocol.

It is also worthwhile mentioning that even though the above societies deemed the use of APBI acceptable in certain patient populations, a joint statement from three German oncology societies recommended refraining from APBI use outside of a prospective study [35]. They felt that there were not enough data available at the time to draw any conclusions about its safety and that it will also decrease the number of women who could be enrolled in ongoing prospective studies [35].

## Controversies in APBI

In 2012, Smith et al. [36] reported that based on their retrospective review of the surveillance, epidemiology, and end results (SEER)-Medicare database, women who underwent APBI between 2003 and 2007 had a higher rate of subsequent mastectomy than those who underwent WBI (3.95% vs. 2.18%, *P* < 0.001). APBI treatment was also more frequently associated with higher rates of infectious and non-infectious postoperative complications, breast pain, fat necrosis, and rib fracture than WBI. The 5-year OS rate however did not differ between the two groups. In a follow-up report, Smith et al. [37] stratified the same population of patients treated with APBI based on the ASTRO consensus guidelines into the suitable, cautionary, and unsuitable groups. Overall, APBI did result in a significant reduction in the risk of mastectomy as compared with lumpectomy alone (2.8% vs. 4.7%). However, the risk reduction was not significant as compared with WBI (2.8% vs. 1.3%). Stratification based on the ASTRO suitability criteria did not show a difference in the relative risk of mastectomy (*P* = 0.84), although suitable patients overall had a low absolute risk of mastectomy after APBI (1.6%).

It is important to keep in mind several limitations of the above studies. The limitations of retrospective studies are self-evident. Furthermore, mastectomy was used as a surrogate end-point since ipsilateral breast tumor recurrence itself is not listed in the SEER-Medicare database and a subsequent mastectomy can be due to a number of reasons unrelated to ipsilateral breast tumor recurrence. Additionally, the period studied (2003–2007) was immediately after the approval of single- and multi-lumen applicators, with many investigators using the device for the first time so it is reasonable to expect relatively higher rates of complications such as infections and fat necrosis.

Another rising area of controversy in breast radiation oncology involves the use of IO-APBI, to the point now that it has garnered national attention as reported on the Wall Street Journal [38]. Some of the controversy surrounds the rising rates of use of IO-APBI given its convenience in one intraoperative fraction and low cost burden to the health care system while questions regarding its safety and efficacy still remain. Some of the issues raised in the ELIOT trial include a significantly higher relapse rate in the IO-APBI group compared with the WBI cohort, no accounting for adverse features on final pathology such as positive margin, higher rates of fat necrosis with IO-APBI, and final cosmesis analysis being performed on only a subset of the patients [39]. The TARGIT-A study has also faced multiple criticisms, such as the excess non-breast cancer deaths and an increased rate of secondary malignancy in the WBI group, both of which usually need much longer-term follow-up to ascertain their significance [39].

Although the points raised in the above studies require consideration, the main questions about safety and efficacy of APBI can only be answered in the ongoing randomized clinical trials.

## Future directions

APBI has now become a mainstay of treatment as part of the BCT algorithm. Even though it’s only a suitable option and should only be considered in patients with early-stage breast cancer based on the consensus statements discussed above, it provides a much faster and convenient alternative to WBI. However, definitive data from randomized controlled trials, which are still ongoing, are needed. The most important of which, NSABP B-39/RTOG 0413 [40] is now closed to accrual. In this trial, WBI to 50–50.4 Gy in 25–28 fractions with an optional 10–16 Gy boost is compared with 34 Gy in 10 fractions delivered either via interstitial brachytherapy, MammoSite^®^, MammoSite ML^®^, or SAVI^®^ or with EB-APBI to a dose of 38.5 Gy in 10 fractions delivered using 3-dimensional conformal radiation therapy. The selection criteria include patients with stage 0, I, or II breast cancer resected by lumpectomy and with no more than three histologically positive nodes. The primary endpoint of the study is time to diagnosis of ipsilateral breast tumor recurrence with secondary endpoints including OS and recurrence-free survival.

There are also other ongoing trials, including a study examining ultrashort-course APBI using Contura^®^ (7 Gy ×4 fractions) [41], the RAPID trial awaiting for final results, release of matured data of the TARGIT and ELIOT studies, and the randomized GEC-ESTRO trial [42]. With most of these trials approaching maturation, the exact role of APBI in the BCT paradigm will be further solidified. Additionally, the appropriate group of patients who can benefit from different APBI modalities will be elucidated, allowing shorter treatment duration, better toxicity profiles, and considerable savings to the health care system [43].
